# Treatment of congenital middle ear cholesteatoma in children using endoscopic and microscopic ear surgeries: a case series

**DOI:** 10.3389/fped.2024.1336183

**Published:** 2024-05-24

**Authors:** Po Xue, Zhaoyan Wang, Yongchuan Chai, Mingjue Si, Lingxiang Hu

**Affiliations:** ^1^ Department of Otolaryngology-Head and Neck Surgery, Shanghai Ninth People’s Hospital, Shanghai Jiao Tong University School of Medicine, Shanghai, China; ^2^ Ear Institute, Shanghai Jiao Tong University School of Medicine, Shanghai, China; ^3^ Shanghai Key Laboratory of Translational Medicine on Ear and Nose Diseases, Shanghai, China; ^4^ Department of Radiology, Shanghai Ninth People's Hospital affiliated to Shanghai Jiao Tong University School of Medicine, Shanghai, China

**Keywords:** cholesteatoma, congenital, endoscopy, middle ear, pediatric

## Abstract

**Introduction:**

Surgical removal is widely employed in children with congenital middle ear cholesteatoma (CMEC). Here, we report the surgical outcomes of CMEC removal via endoscopic ear surgery (EES) and microscopic ear surgery (MES) in children.

**Methods:**

Children with CMEC who underwent preoperative medical history inquiry, hearing test, endoscopic evaluation, and radiology imaging before receiving EES or MES were included. Postoperative audiological outcomes and recurrence rates were collected.

**Results:**

Seventeen children (20 ears) with stage II-IV CMEC were included. Of those, 11 ears (55.0%) underwent EES, and 9 ears (45.0%) underwent MES. The follow-up time was 35 ± 13.5 months. One child in the EES group with stage III CMEC had a recurrence during the follow-up period. In the EES group, the average minimum diameter of the external auditory canal on the affected side was 5.8 mm (4.3–8.0 mm). No linear association was found between age and the minimum diameter of the external auditory canal.

**Discussion:**

EES is a promising treatment option for children with early-stage CMEC because of its low recurrence rate and minimally invasive nature. The minimum diameter of the external auditory canal on the affected side should be meticulously examined when performing EES in children.

## 1 Introduction

Congenital middle ear cholesteatoma (CMEC) is defined as a white, pearly mass with keratinized squamous epithelium located behind an intact tympanic membrane (TM) in the absence of prior surgical procedures or perforation, which is most prevalent in young patients (1, 2). The pioneering work of Derlacki EL, Clemis JD proposed the residual epithelium hypothesis, diagnostic criteria, and classification of congenital cholesteatoma (3). As CMEC progresses, hearing loss, otorrhea, and obstruction of the eustachian tube may result in secretory otitis media, and repeated purulent discharge may occur after TM penetration. Further development can result in facial nerve paralysis, labyrinthine fistulas, and even intracranial complications (4). CMEC is more aggressive in children than in adults (5). The early onset of CMEC may have no clinical manifestations, and delays in intervention may facilitate the extension of the disease (6). Thus, early surgical treatment is warranted, as CMEC progression is closely related to patient age.

Surgical removal of cholesteatomahighly relies on visualization of the full extent of the disease. However, given the small size of the pediatric external auditory canal, the anatomy of the middle ear can be challenging to visualize and approach via the ear canal in children (7). Microscopic ear surgery (MES) has been widely used in the past, but the residual recurrence rate is higher than in adults (8). In recent years, promising results have been achieved with the increasing use of endoscopes in the surgical treatment of adult cholesteatoma (9, 10). Compared with MES, endoscopy allows for a wider viewing angle with an increased depth of field and improved resolution with magnification, which is particularly helpful when navigating the middle ear space (11). Studies have investigated the endoscopic treatment of cholesteatoma (12, 13). However, evidence for the feasibility and clinical efficacy of endoscopic ear surgery (EES) for pediatric cholesteatoma remains limited and warrants further exploration. Additionally, the scope of view in the external auditory canal mainly depends on the minimum diameter of the narrowest segment of the ear canal, which limits the application of EES in clinical practice. Due to the relatively narrow minimum diameter of the pediatric external auditory canal, the patient's age and the minimum diameter of the ear canal have become crucial practical considerations for EES for surgeons. As such, surgeons are mainly concerned with determining the appropriate conditions and anatomical features required to perform ESS on pediatric patients. For congenital cholesteatoma, a staging system was proposed by Potsic et al. (2002) that divides congenital cholesteatoma into four stages: stage I, lesions in a single quadrant of the TM without involvement of the ossicular chain or mastoid process; stage II, lesions in multiple quadrants without invading the ossicular chain or mastoid process; stage III, lesions in multiple quadrants that invade the ossicular chain without involvement of the mastoid process; and stage IV, lesions in multiple quadrants that develop into the mastoid process (14). Previous studies have shown a correlation between cholesteatoma stage and the choice of surgical approach and postoperative recurrence rate (15, 16). Early surgery with correct staging and selection of an appropriate surgical approach helps to reduce surgical trauma and improve the prognosis. For these reasons, this study retrospectively reports a series of pediatric patients with CMEC who underwent EES or MES to provide a clinical reference for CMEC management in children.

## 2 Materials and methods

This study retrospectively reviewed a series of pediatric patients with CMEC who were treated at the Department of Otorhinolaryngology at our hospital from November 2018 to February 2022. The inclusion criteria were as follows: (1) age <18 years old; (2) preoperative audiological evaluation with pure-tone audiometry; (3) preoperative endoscopic evaluation; (4) preoperative high-resolution temporal bone computerized tomography (CT) evaluation confirming CMEC diagnosis; and (5) follow-up time >6 months. The exclusion criteria were as follows: (1) an incomplete preoperative evaluation and (2) failure to return to the unit for follow-up. The study was approved by the ethics committee of our hospital (approval number: SH9H-2023-T103-1). Informed consent was obtained from the parents of the patients.

### 2.1 Preoperative evaluation

All children underwent a detailed preoperative medical history inquiry and endoscopic evaluation of the TM. The criteria for CMEC evaluation were as follows: (1) the edge of the TM was intact without apparent indentation; (2) there was no continuity between the epithelium of the cholesteatoma and the TM; and (3) there was no history of ear surgery or repetitive otitis media. The pure-tone threshold average and the air-bone gap were calculated using the thresholds of 0.5, 1, 2, and 4 kHz. High-resolution temporal bone CT was performed to evaluate the extent of the disease and measure the minimum diameter of the external auditory canal.

### 2.2 Surgical procedures

For children who met the inclusion criteria, the same surgeon performed all EES procedures using a transcanal approach. The surgery was performed under general anesthesia with the patient in the supine position with their head tilted 45° toward the healthy side. A 0° angle ear endoscope with a 2.7-mm outer diameter was used. The main process was as follows: (1) After the patients were subcutaneously injected with 1:20,000 epinephrine into the external auditory canal (3–5 min), a circumferential incision was made approximately 0.8 cm from the tympanic annulus at 12 to 6 o’clock in the skin of the external auditory canal to elevate the TM flap and expose the facial nerve. (2) The posterior wall of the external auditory canal was resected as needed to expose the cholesteatoma fully. (3) The cholesteatoma was thoroughly dissected along the edge while protecting the facial nerve and ossicles. (4) If ossicular chain defects were noted during surgery, a concomitant ossiculoplasty using an artificial total or partial ossicular replacement prosthesis (TORP or PORP) may also be performed. When stapes were present and mobile, a PORP was performed. When the footplate superstructure was absent, a TORP was prepared. (5) A piece of cartilage with soft tissue taken from the tragus cartilage was used to reconstruct the posterior wall of the external auditory canal. If the patient had a perforation, graft tissue was placed on the undersurface of the TM flap to reconstruct the TM. (6) The TM flap was returned to its original position. (7) Finally, the middle ear and external auditory canals were filled with antibiotics and packed with an absorbable ear dressing.

### 2.3 Postoperative follow-up

Intravenous antibiotics were administrated postoperatively. The children were discharged on the third day after surgery. All children were asked to take antibiotics for one week after discharge. Regular follow-ups were scheduled at 2 weeks, 1 month, and 3 months with an ear endoscopy. An audiology test was conducted during the 3-month follow-up. For patients without any special symptoms, an annual follow-up was conducted. During the endoscopic examination, an audiology test and high-resolution temporal bone CT would be promptly performed if there were any symptoms such as hearing loss, otorrhea, or abnormal TM.

### 2.4 Outcome evaluation

**Figure 1:**
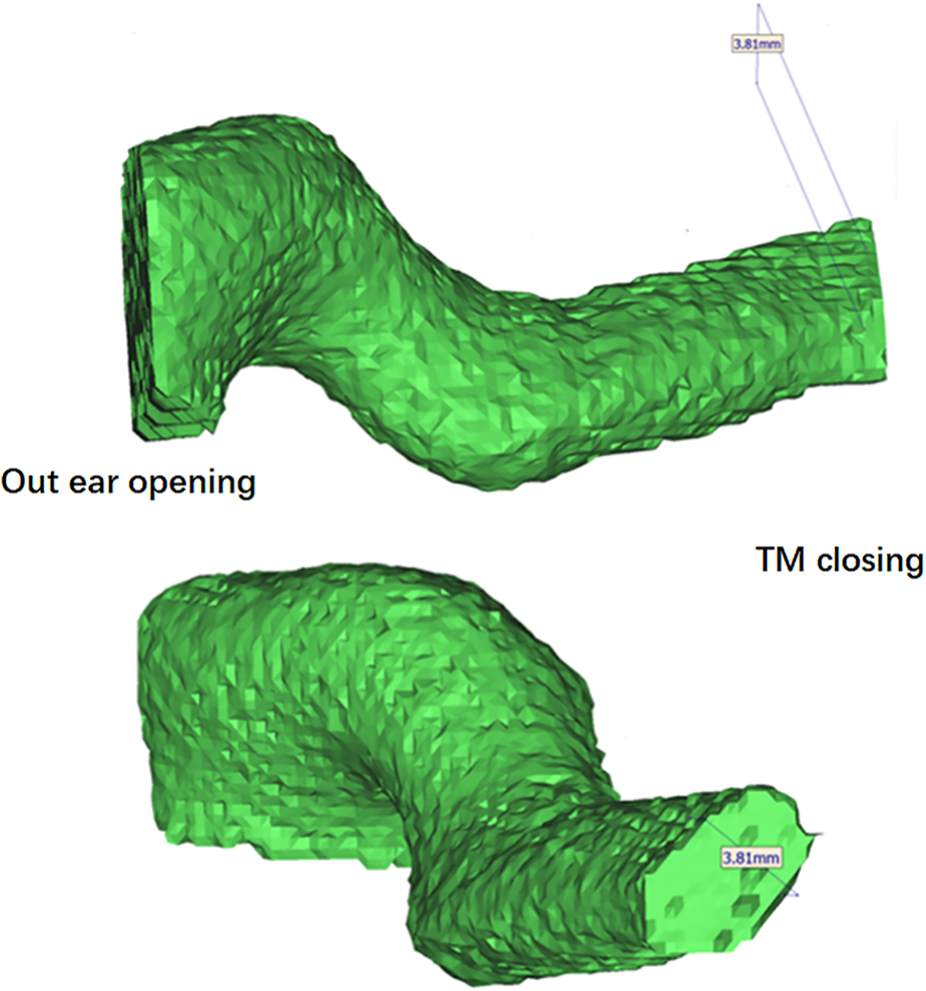
3d modeling of the external auditory canal using mimics research 21.0 to measure the minimum diameter.

A retrospective medical record review and data collection were conducted by an independent reviewer (Lingxiang Hu) who was not involved in the surgeries or postoperative follow-up. The minimum diameter of the external auditory canal in the narrowest segment was measured using CT imaging employing 3D modeling (Mimics Research 21.0, Figure 1).

## 3 Results

**Figure 2:**
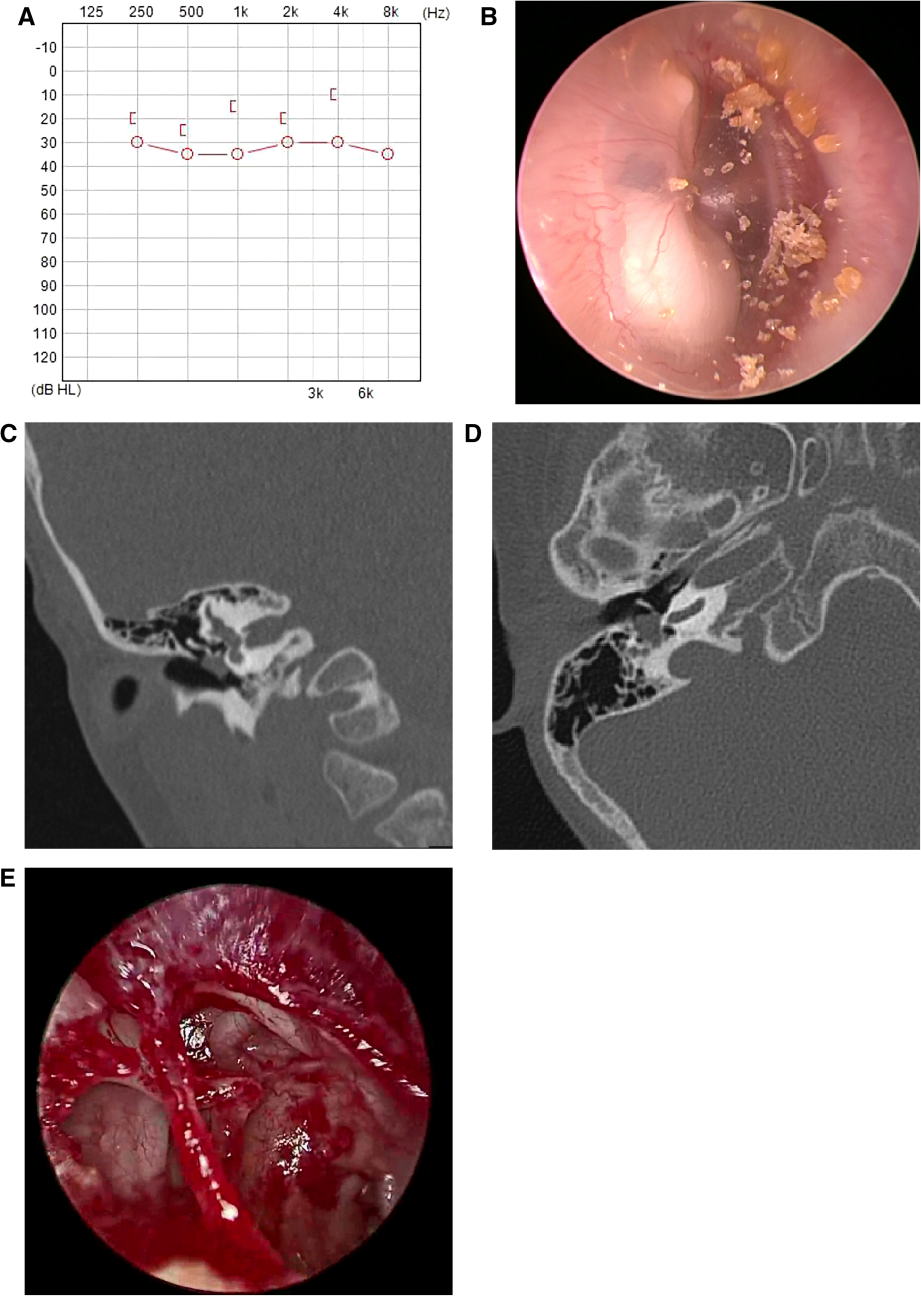
Typical case of stage II CMEC. (**A**) An audiogram revealed mildly abnormal hearing before surgery; (**B**) Endoscopic findings revealed a white mass involving the post superior and inferior quadrants of the tympanic membrane; (**C,D**) CT showing soft tissue density in the right side of the middle ear, without involving the mastoid; (**E**) After the complete removal of the congenital cholesteatoma, the ossicular chain was intact.

**Figure 3:**
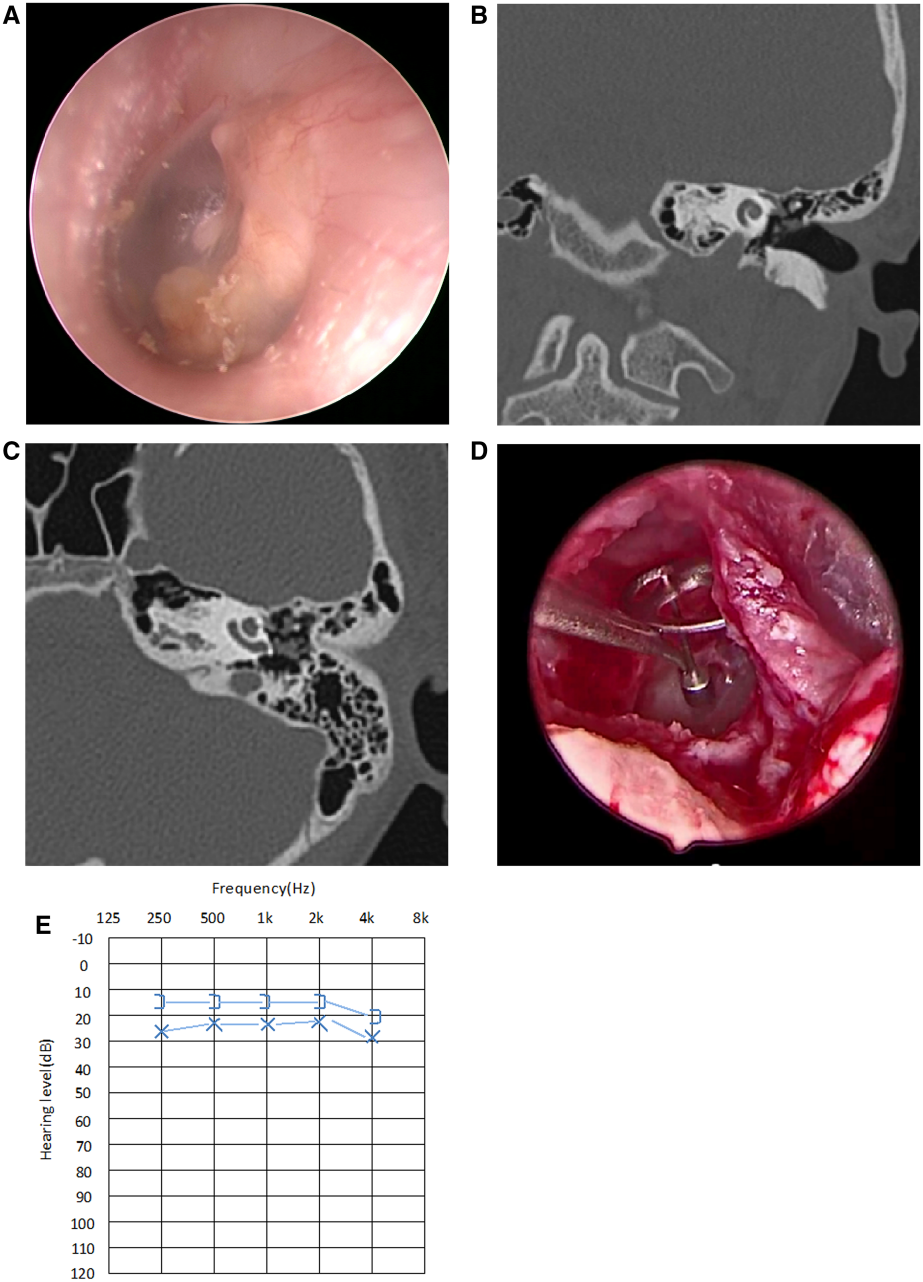
Typical case of stage III CMEC. (**A**) Endoscopic findings revealed a white mass involving multiple quadrants of the tympanic membrane; (**B,C**) CT showed a soft tissue density in the left side of the middle ear, with the ossicular chain impaired, but without involving the mastoid; (**D**) After complete removal of the congenital cholesteatoma, the stapes superstructure was absent, so TOPR was conducted for hearing reconstruction; (**E**) Postoperative audiogram at the 3-month follow-up showed a mild conductive hearing impairment, with 26.3 dB HL in air conductance, 16.3 dB HL in bone conduction, and 10 dB HL in the air-bone gap.

Seventeen children (including 20 ears from 11 males and 6 females) were included in the study, with an average age of 10 ± 3.6 years at the time of surgery. Based on Potsic staging, 1 ear (5.0%) was in Potsic stage II (Figure 2), 10 ears (50.0%) in Potsic stage III (Figure 3), and 9 ears (45.0%) in Potsic stage IV. The average minimum diameter of the external auditory canal on the affected side was 5.8 mm, with a range of 4.3–8.0 mm. Among the 20 ears, 11 (55.0%) underwent EES through the external auditory canal. Nine ears (45.0%) underwent MES. Four ears (20.0%) did not undergo ossiculoplasty during surgery, 6 ears (30.0%) underwent PORP, and 10 ears (50.0%) underwent TORP. The average follow-up was 35 ± 13.5 months (all ears were dry 3 months after surgery). No recurrence was observed in 19 ears (95.0%), while one child with stage III CMEC (left side of case 17) had a recurrence after 1 year of follow-up. This child underwent a second endoscopic surgery through the ear canal, and no recurrence was observed after 17 months of follow-up. During the follow-up period, none of the children experienced any complications, such as sensorineural hearing loss, dizziness, facial paralysis, or implant displacement (Table 1). Figures 2, 3 show the preoperative evaluation and postoperative outcomes of two representative cases.

**Table 1:** Preoperative demographic and postoperative audiometric results of 17 children (20 ears).

Case	Gender	Age/month	Side	Narrowest diameter (affected side)	Narrowest diameter (unaffected side)	Cholesteatoma range (under endoscope)	Stage	Surgery method	Hearing reconstruction	Pre-/post-surgery hearing (dB)	Followed-up visit(M)
1	M	194	Left	4.8	4.4	ASQ&PSQ	IV	CWUT	PORP	52/38.8	48
2	F	44	Right	5.3	4.7	AIQ&ASQ	III	Endoscope	N/A	50/37	35
3	M	136	Right	7.2	7.9	PIQ&PSQ	III	Endoscope	N/A	31/21	46
4	M	55	Left	6.2	5.7	PIQ&PSQ	IV	CWUT	TORP	48.8/40	44
5	M	118	Right	6.4	5.5	ASQ&AIQ	IV	CWUT	TORP	32.5/31	44
6	F	133	Left	5.4	5.8	ASQ&PSQ	IV	CWUT	TORP	61/37.5	43
7	M	211	Left	3.8	4.4	ASQ&PSQ	IV	CWDT	PORP	50/37.5	51
8	M	120	Left	7	5.8	PIQ&PSQ	II	Endoscope	N/A	32.5/25	27
9	M	58	Right	4.8	5.9	ALL	IV	CWUT	TORP	32/33.8	18
10	F	84	Left	5.8	5.6	ASQ&PSQ	III	Endoscope	PORP	21/12.5	31
11	M	64	Right	4.7	5.1	AIQ&ASQ	III	Endoscope	TORP	31/40	15
12	M	147	Left	4.3	5.5	AIQ&ASQ	III	Endoscope	TORP	45/36.3	15
13	M	135	Left	5	4.8	PIQ&PSQ&AIQ	III	Endoscope	TORP	37.5/26	22
14	F	77	Left	7.3	5.6	PSQ&PIQ	IV	CWUT	TORP	58/41.3	22
15	M	100	Left	4.9	/	AIQ&PIQ	IV	CWUT	N/A	45/33.8	28
15	M	106	Right	4.1	/	PSQ	IV	CWDT	TORP	48/40	22
16	F	134	Right	8	/	AIQ&PIQ	III	Endoscope	PORP	40/31.3	53
16	F	137	Left	5.3	/	ASQ&PSQ	III	Endoscope	PORP	35/37.5	51
17	F	144	Right	5.3	/	PSQ	III	Endoscope	PORP	55/38.8	54
17	F	148	Left	5.8	/	PSQ&PIQ	III	Endoscope	TORP	61/41	51

ASQ, anterior superior quadrant of the TM; AIQ, anterior inferior quadrant of the TM; PSQ, posterior superior quadrant of the TM; PIQ, posterior inferior quadrant of the TM; ALL, entire TM; PORP, partial ossicular replacement prosthesis; TOPR, total ossicular replacement prosthesis; CWUT, canal wall-up tympanoplasty; CWDT, canal wall-down tympanoplasty.

## 4 Discussion

This study retrospectively analyzed 17 children (20 ears) with stage II–IV CMEC who underwent either EES or MES and were followed up for 27 months after surgery. The results showed only one ear with stage III CMEC who received EES had a recurrence after 1 year of follow-up. Compared to MES, EES has a broad range of applications and has achieved positive results owing to its ability to enhance hard-to-visualize areas of the middle ear.

EES has currently been widely used for middle ear surgery in the adult ear canal (17). However, it is essential to consider the narrower ear canal in children when opting for EES during auditory surgery. The minimum diameter of the narrowest segment of the ear canal is the main factor that limits EES application through the ear canal (9, 10). A previous study (18) included 16 children and 35 adults and showed that the minimum diameter of the narrowest segment of the ear canal in children was 3.6–5.9 mm and the maximum diameter was 6.5–10.2 mm. In adults, the minimum diameter of the narrowest segment of the ear canal was 3.4–6.6 mm and the maximum diameter was 6.9–15.0 mm. Although children's ear canals are generally narrower than adult ear canals, there was no significant difference in the minimum diameter of the narrowest segment between children and adults, which suggests the feasibility of using EES among children.

Ghadersohi et al. (19) successfully resected a 14-month-old's congenital cholesteatoma via a transcanal endoscopic approach. In a study by Park et al. (9), the youngest patient was 17 months old, and the cholesteatoma was removed without difficulty via an entirely transcanal endoscopic approach with a 3.0-mm rigid endoscope. Kobayashi et al. (13) successfully performed total endoscopic ear surgery (TEES) on a 23-month-old male infant with an external auditory canal diameter of 5.6 mm. They considered that an external auditory canal diameter of more than 4.5 mm on CT was required for the performance of TEES using endoscopes 2.7 mm in diameter. The diameter was measured by the minimum distance between the posterior and anterior bony wall of the ear canal at the middle point of the entrance to the ear canal and the umbo. Ear canals smaller than 4.5 mm in diameter can be accessed with a postauricular incision, with or without drilling of the external ear canal. In these studies, the age and canal diameter of patients with normal external auditory canals did not critically affect the EES results. In an anatomical study (20), the median minimum diameter of the ear canal was 5.45 mm (IQR = 1.67) and all specimens achieved complete intraoperative exposure of middle ear landmarks via endoscopy. James et al. (21) suggested that in many cases, the curvature of the ear canal rather than the child's age controls endoscopic access. Kim et al. (22) suggested that a 0° endoscope has some limitations in the viewing angle; therefore, an angular range of at least 30° or greater is required for successful detection. To prevent hitting the malleus handle or short process, the endoscope is introduced into the middle ear cavity with its beveled angle facing the malleus. Additionally, a 4-mm-diameter endoscope can improve the probability of hitting the malleus even with advancement instructions. Thus, the use of endoscopes with diameters <3 mm will help prevent accidental collisions.

**Figure 4:**
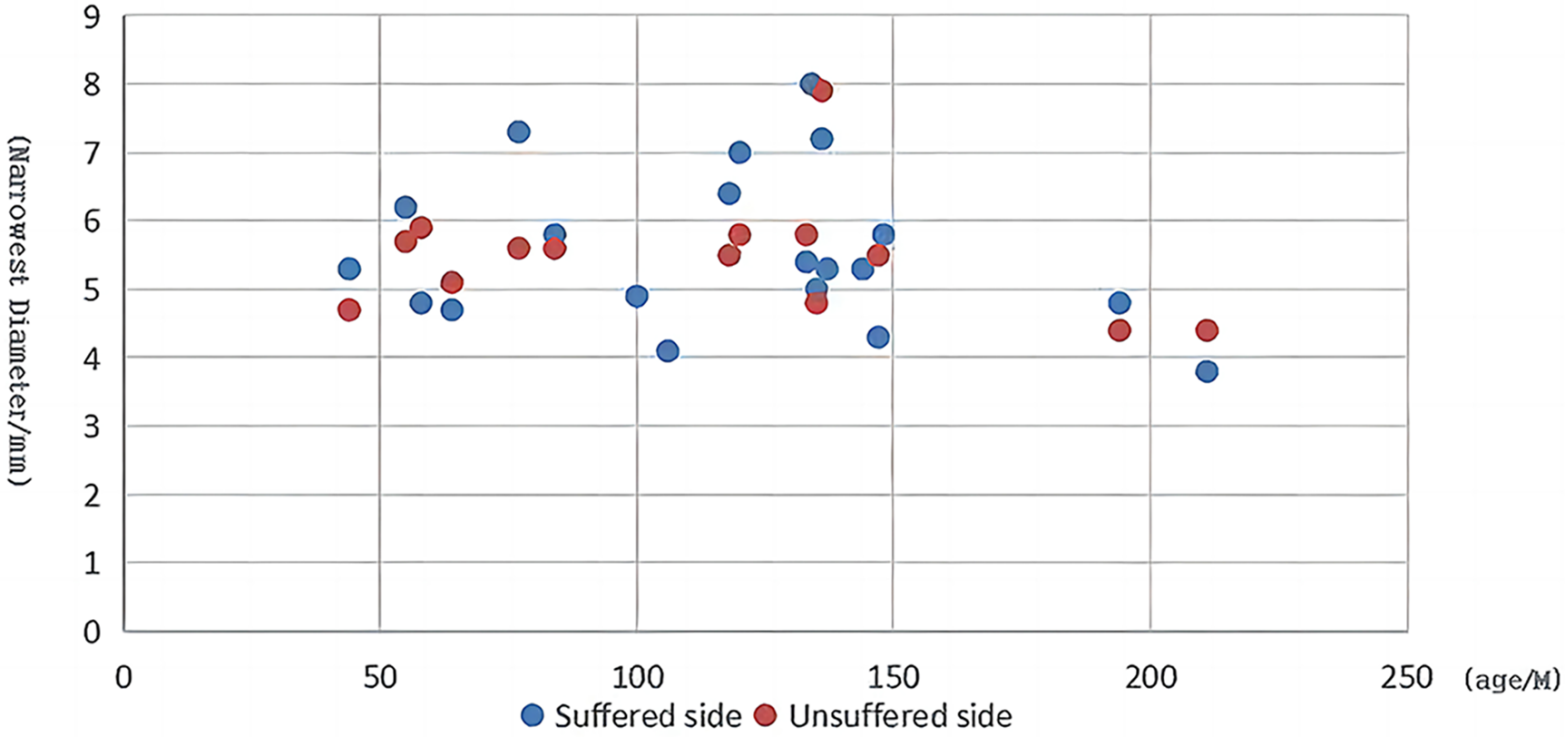
Scatter plot of child age vs. the minimal diameter at the narrowest segment of the ear canal.

Furthermore, with the evolution of endoscopes and other instruments, the obstacles to applying EES in the relatively narrow ear canal among children have now been overcome, and EES is emerging as an option in pediatric middle ear surgery. In our study, the range of the minimum diameter of the ear canal at the affected side was 4.3–8 mm in the EES group. Moreover, we did not observe a linear relationship between the minimum diameter and age (Figure 4), plausibly indicating that age is not a key factor limiting EES application.

Conventional MES includes canal wall-up tympanoplasty (CWUT) and canal wall-down tympanoplasty (CWDT) (15). To protect hearing and reduce damage, CWUT is often used to treat cholesteatoma among children (23). However, conventional CWUT has a relatively limited field of view, especially when conducted in children, as well as a relatively high residual rate after surgery. Compared to MES, endoscopy provides a wide field of view and offers enhanced visualization to inspect middle ear ventilation pathways for blockage and potentially hidden areas. Previous studies have shown that endoscopic treatment in CMEC has a lower residual or recurrence rate (12, 24, 25). Hunter et al. (26) retrospectively analyzed 76 patients with cholesteatoma who underwent TEES and microscopic surgery with a follow-up of 3.5 years. The rate of residual disease after TEES was 20.0%, whereas the rate of residual disease after microscopic surgery was 40.0%. Another study (27) retrospectively reviewed 59 patients (no mastoid involvement, Potsic stage I–III) with a follow-up time of 3.2 years. They reported that the rate of residual disease after endoscopic surgery was 19.3%, whereas the rate of residual disease after microscopic surgery was 34.4%. The results of these previous studies indicate that patients who underwent microscopic surgery might be exposed to a higher residual or recurrence rate than those who underwent endoscopic surgery for the treatment of congenital cholesteatoma. Similarly, our results demonstrated a relatively low recurrence rate (9%), suggesting that EES has promising surgical outcomes in clinical treatment for children with CMEC. Despite the prominent outcome, these studies barely reported or analyzed the minimum diameter of the external auditory canal of their patients, and difficulties in surgical treatment were still experienced by children with CMEC due to ear canal stenosis. The diameter of the isthmus of the external auditory canal is critical when practicing EES. The cases in our study who underwent EES reported an average diameter of 5.8 mm on the affected side, with the lowest diameter of 4.3 mm, suggesting that EES was feasible for these children. These results provide a reference for surgeons in decision-making for surgical procedures among children with CMEC.

**Figure 5:**
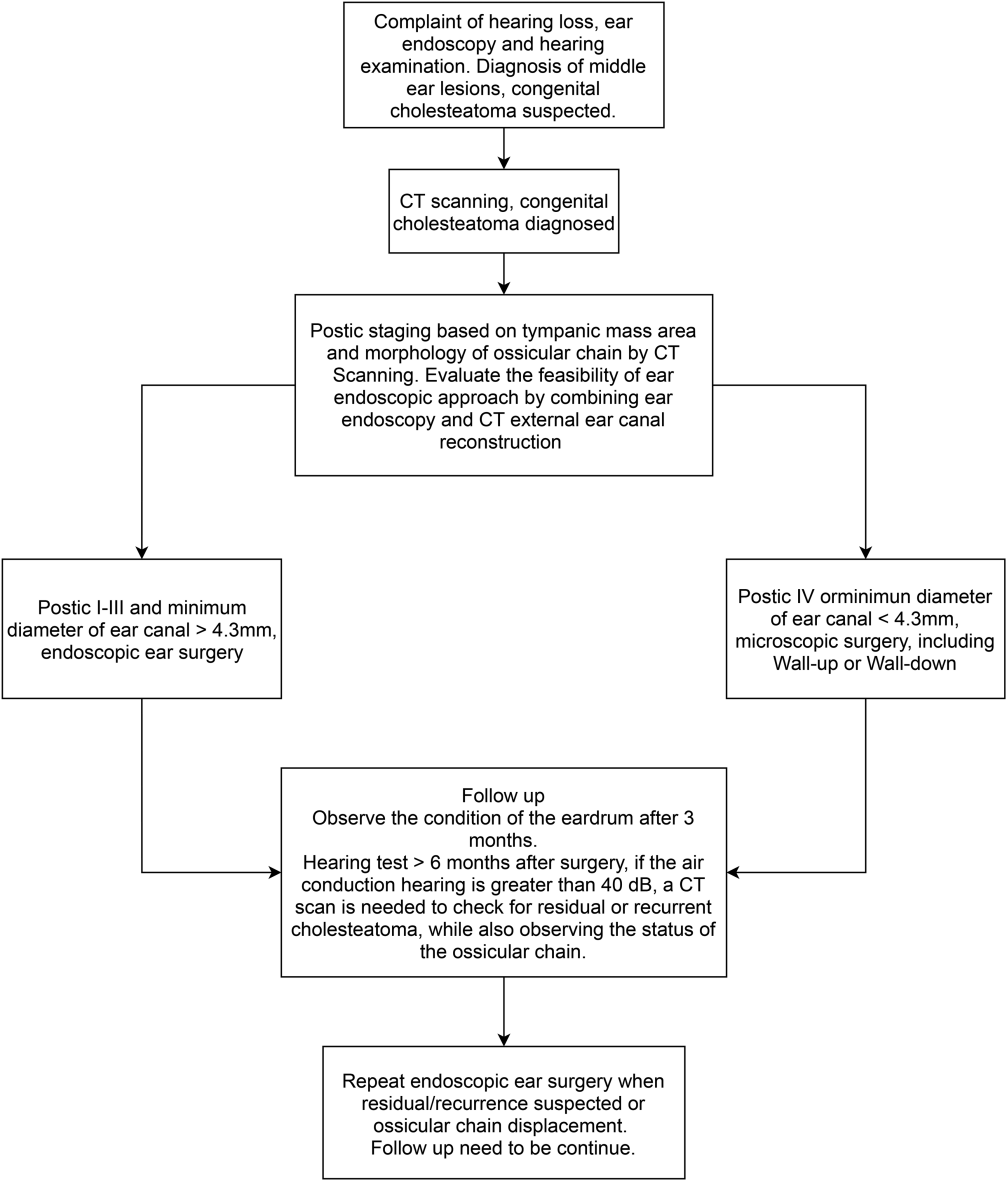
The decision-making process of choosing between EES and MES for pediatric CMEC.

The surgical treatment of CMEC with an endoscope may achieve good results, but it may be less suitable for some patients. Park (9) retrospectively analyzed 25 cases of CMEC in children, of whom 20 were in Potsic stage I and II and one case had postoperative recurrence. The remaining five children were in Potsic stage III with no recurrence. The author claimed that EES was unsuitable for children in Potsic stage IV, so they were not included in their study. A recently published study (10) retrospectively included 115 patients with CMEC who received different surgical approaches based on their Potsic stages. After a 2-year follow-up, 58 patients who were diagnosed with Potsic stage I and II and received EES reported a recurrence rate of 1.7% (1/58). Furthermore, 24 patients who were diagnosed with Potsic stage III reported a recurrence rate of 12.5% (3/24) under endoscopic surgery. Twenty-eight patients who were diagnosed with Potsic stage IV and underwent MES reported a recurrence rate of 21.4% (6/28). Based on previous literature and our findings, EES is highly recommended for children in Potsic stage I–III due to the low residual and recurrence rate and the aesthetic advantage (less invasive). For children in Potsic stage IV, we believe conventional MES or endoscope-assisted microscopic surgery is more appropriate. Future studies should consider a multi-centered prospective approach with a sufficient sample size to further explore the efficacy of both surgical approaches in children with different stages of CMEC. Previous studies regarding otoendoscopic approaches for treating congenital cholesteatoma in children are summarized in Table 2 (4, 9, 12, 13, 19, 22, 25–29). We also propose a simple flowchart to facilitate the decision-making process of choosing between EES and MES for pediatric CMEC (Figure 5).

**Table 2:** Summary of 11 studies of total endoscopic ear surgery.

Author/year	Study design	Ears	Age/year	F/U period/month	Postic stage				CC/mm	Endoscopes/mm/cm	Pre-surgery hearing/dB	Post-surgery hearing/dB
					Postic-I	Postic-II	Postic-III	Postic-IV				
Kobayashi et al. (12)	retrospective case review	12	3 (1–6)	23.1	7 (ASQ)	4 (3ASQ + 1PSQ)	1 (PSQ,1 residual)	0	3.9 (2.8–5.7)	2.7/11 (0°/30°), 1.9 (30°)	4 ears, no any deterioration	7 ears, 12.7 (3.3–23.9)
Marchioni et al. (26)	retrospective case review	31	9.6 (4–16)	36 (8–88)	/	/	/	/	/	/	PORP: 28.9, TORP: 47.5	PORP: 28.9, TORP: 47.5
Hunter et al. (25)	retrospective case review	8	11.6 (5–17)	15.3 (7–26)	/	/	/	/	/	/	27.4	16.7
		21 (combined endoscopic)	11 (6–16)	13.8 (7–24)	/	/	/	/	/	/	33.9	24.3
Ghadersohi et al. (18)	retrospective case review	10	3.8 (1.2–6.8)	25.2 (9.6–42)	/	/	/	/	/	3.0 (0°/30°/45°/70°)	11.6	20.8
Kim et al. (21)	retrospective case review	3	3.67 (2–10)	15.5 (3.5–38)	3	0	0	0	/	2.7/14 (30°)	16.6 ± 3.34	16.3 ± 3.27
		9 (combined endoscopic)			5	2	2	0	/			
Park et al. (8)	retrospective case review	25	3.8 (1.4–9)	24 ± 8.5	13	7 (1 recurrence)	5	0	3.8 (0.5–7.6)	3.0 (0°/30°), 3.0 (45°)	20 ears, 17.7 (6.3–53.8)	21 ears, 18.9 (6.3–43.8)
Hao et al. (3)	retrospective case review	29	/	60	13	10	6	0	/	/	/	/
Choi et al. (11)	retrospective case review	21	3.1 ± 0.95	22.02 ± 13	16 (2 recurrence)	1	4	0	3.2 ± 1.7 (2.5–8.5)	3.0 (0°/30°)	/	/
Zeng et al. (24)	retrospective case review	11	7.91 ± 3.11 (3–14)	(6–12)	3	4	4	0	/	3.0 (0°/30°)	36.19 ± 10.75	17.84 ± 14.67
Jang et al. (27)	retrospective case review	46	3 ± 2.6 (1–17)	27.3 ± 19.2	35	5	6	0	3.9 ± 2.0 (1.2–13)	2.7/14 (0°/30°), 3.0/14 (45°)	/	/
Choi et al. (28)	retrospective case review	271	3.5 ± 2.9	29.7 ± 22.2	190 (15 residual)	21 (3 residual)	57 (18 residual)	3	/	/	27.3	23.2

F/U, follow up; CC, congenital cholesteatoma; RCR, residual cholesteatoma or recurrence.

.

Our study has some limitations. First, its retrospective design may introduce biases in patient selection and data collection. Second, our small sample size and short follow-up time may limit the representativeness of our findings. Third, the lack of enhanced MRI examination in our study may reduce the diagnostic capability for CMEC recurrence.

In conclusion, EES is a clinically promising option for the treatment of children with CMEC, and it is especially recommended for children in Potsic stages I–III owing to the low residual and recurrence rates and minimally invasive nature. For children in Potsic stage IV, MES or endoscope-assisted microscopic surgery should be adopted. The minimum diameters of the external auditory canal on the affected side should be meticulously measured when performing EES in children. Detailed audiology tests, endoscopic examinations, and radiology imaging should be performed preoperatively for children with CMEC to assess the extent of the lesion for clinical treatment.

## Data Availability

The raw data supporting the conclusions of this article will be made available by the authors, without undue reservation.
